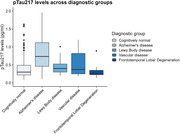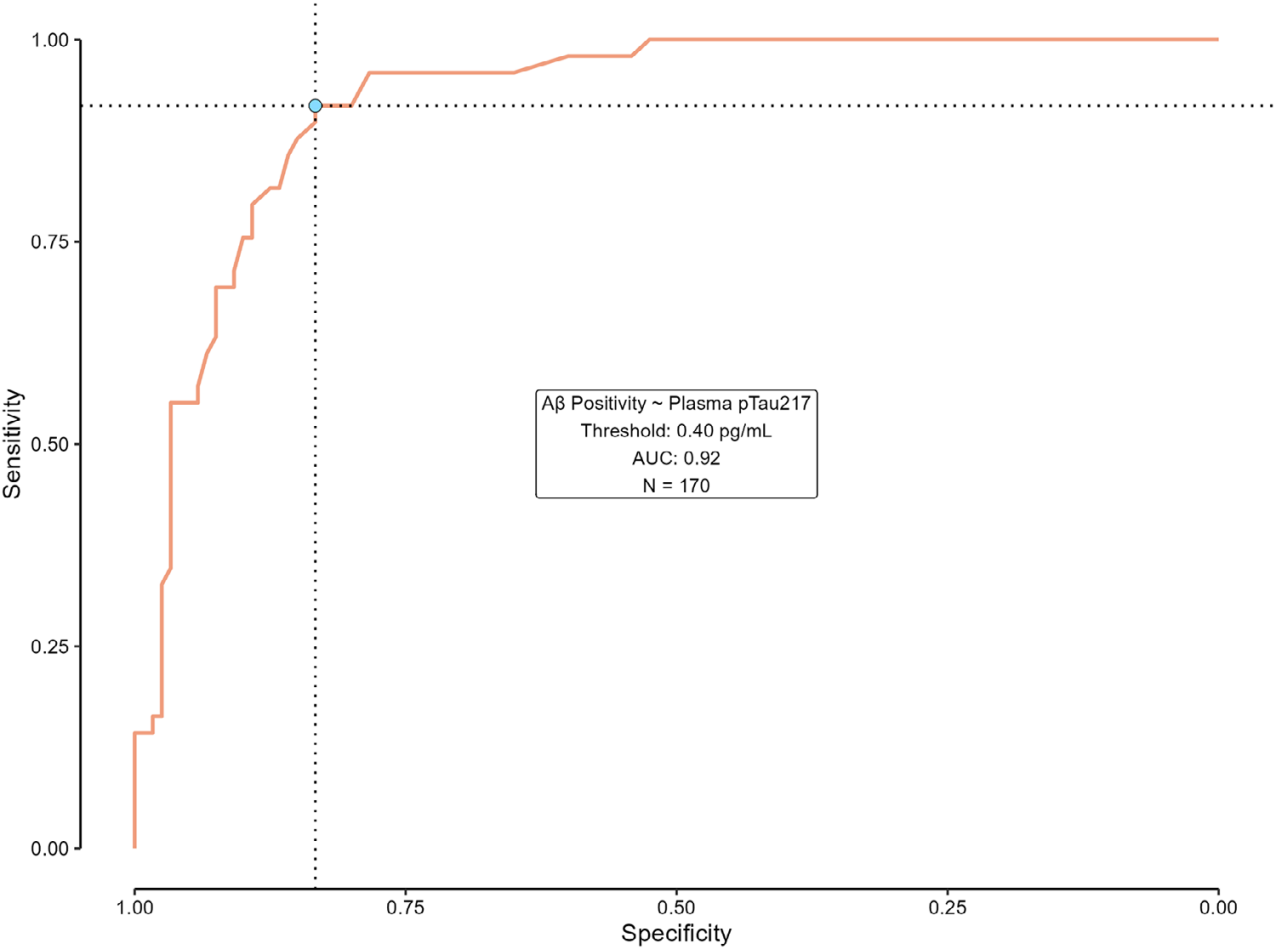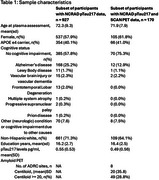# Integration of plasma and imaging data within the ADRC biofluid and imaging ecosystems

**DOI:** 10.1002/alz70856_104199

**Published:** 2026-01-12

**Authors:** Julie E. Oomens, Theresa M. Harrison, Jeffrey L. Dage, Kristen A. Russ, Henrik Zetterberg, William J. Jagust, Tatiana M. Foroud, Sarah Biber, Elizabeth C. Mormino, Sterling C Johnson

**Affiliations:** ^1^ Wisconsin Alzheimer's Disease Research Center, University of Wisconsin School of Medicine and Public Health, Madison, WI, USA; ^2^ Neuroscience Department, University of California, Berkeley, Berkeley, CA, USA; ^3^ Department of Medical and Molecular Genetics, Indiana University School of Medicine, Indianapolis, IN, USA; ^4^ Department of Neurology, Indiana University School of Medicine, Indianopolis, IN, USA; ^5^ Stark Neurosciences Research Institute, Indiana University School of Medicine, Indianapolis, IN, USA; ^6^ Indiana Alzheimer's Disease Research Center, Indianapolis, IN, USA; ^7^ Hong Kong Center for Neurodegenerative Diseases, Hong Kong, Science Park, China; ^8^ Department of Psychiatry and Neurochemistry, Institute of Neuroscience and Physiology, the Sahlgrenska Academy, University of Gothenburg, Molndal, Sweden; ^9^ Wisconsin Alzheimer's Disease Research Center, University of Wisconsin‐Madison, School of Medicine and Public Health, Madison, WI, USA; ^10^ Department of Neurodegenerative Disease, UCL Institute of Neurology, London, United Kingdom; ^11^ UK Dementia Research Institute, University College London, London, United Kingdom; ^12^ Clinical Neurochemistry Laboratory, Sahlgrenska University Hospital, Mölndal, Västra Götaland län, Sweden; ^13^ Department of Epidemiology, School of Public Health, University of California, Berkeley, CA, USA; ^14^ National Alzheimer's Coordinating Center, University of Washington, Seattle, WA, USA; ^15^ Department of Neurology and Neurological Sciences, Stanford University, Stanford, CA, USA; ^16^ Wisconsin Alzheimer's Institute, University of Wisconsin School of Medicine and Public Health, Madison, WI, USA

## Abstract

**Background:**

Data accessibility and interoperability across the U.S. Alzheimer's Disease Research Centers (ADRCs) provides necessary resources and data access to enable novel hypothesis testing without additional data collection and will allow end users to rapidly advance our understanding of multiple pathologies or multiple chronic conditions on disease progression within Alzheimer's disease and related diseases. The aim of the current study was to integrate plasma data from the National Centralized Repository for Alzheimer's Disease and Related Dementia's (NCRAD) with ADRC neuroimaging data from the SCAN initiative and National Alzheimer's Coordinating Center Uniform Data Set (NACC UDS) demographic data, all publicly available through the NACC Data Platform and Data Front Door. We provide sample descriptives and present the results of initial data explorations.

**Method:**

The NACC and NCRAD data request procedures were completed to gain access to the data. We focused on the subset of participants for whom Quanterix Simoa HD‐X Alzpath plasma pTau217 data was available (NCRAD). Amyloid pathology was defined based on centiloid values (cut‐off >= 20; SCAN initiative). Demographic information was available for all participants (NACC UDS). We integrated data using the NACC identifier and visit age where available. We used Spearman correlations to assess the association between plasma pTau217 and centiloid values and we used ROC analyses to assess amyloid classification performance.

**Result:**

Plasma pTau217 data was available for 927 participants (sample descriptives in Table 1). Figure 1 shows the distribution of plasma pTau217 levels across diagnostic groups. In the subset of participants for whom amyloid PET was available (*n* = 170, Table 1), the Spearman correlation between plasma pTau217 levels and centiloid values was 0.59. Quanterix pTau217 accurately classified amyloid status with a ROC AUC of .92 (95%CI 0.89–0.97; accuracy 85%; Figure 2). Tau PET was available for 135 participants with plasma pTau217 data and 114 participants with both plasma pTau217 and amyloid PET data.

**Conclusion:**

Combining the SCAN PET and NCRAD plasma results in a promising resource, already with 36% demographic diversity. Sample sizes will increase through ongoing efforts as part of the SCAN initiative and the ADRC Consortium for Clarity in ADRD Research Through Imaging (CLARiTI).